# Supplementary material for: Ferric reduction by a CYBDOM protein counteracts increased iron availability in root meristems induced by phosphorus deficiency
Source: Nat Commun. 2024 Jan 11;15:422. doi: 10.1038/s41467-023-43912-w (PMC10784544; doi:10.1038/s41467-023-43912-w)
Supplement: Supplementary file 1 — Supplementary Information [file 41467_2023_43912_MOESM1_ESM.pdf]

Supplementary information for

Ferric reduction by a CYBDOM protein counteracts increased iron availability in root meristems induced by phosphorus deficiency

Rodolfo A. Maniero[1], Cristiana Picco[2], Anja Hartmann[1], Felipe Engelberger[3], Antonella Gradogna[2], Joachim Scholz-Starke[2], Michael Melzer[1], Georg Künze[3,4,5], Armando Carpaneto[2,6], Nicolaus von Wirén[1], Ricardo F.H. Giehl[1]*

[1] Leibniz Institute of Plant Genetics & Crop Plant Research (IPK) OT Gatersleben, Corrensstr 3, 06466 Seeland, Germany.

[2] Institute of Biophysics, National Research Council, Via De Marini 16, 16149, Genoa, Italy.

[3] Institute for Drug Discovery, Leipzig University, SAC 04103, Leipzig, Germany.

[4] Center for Scalable Data Analytics and Artificial Intelligence, Leipzig University, 04105 Leipzig, Germany.

[5] Interdisciplinary Center for Bioinformatics, Leipzig University, 04107 Leipzig, Germany.

[6] Department of Earth, Environment and Life Sciences (DISTAV), University of Genoa, Viale Benedetto XV 5, 16132, Genoa, Italy.

* To whom correspondence may be addressed: giehl@ipk-gatersleben.de

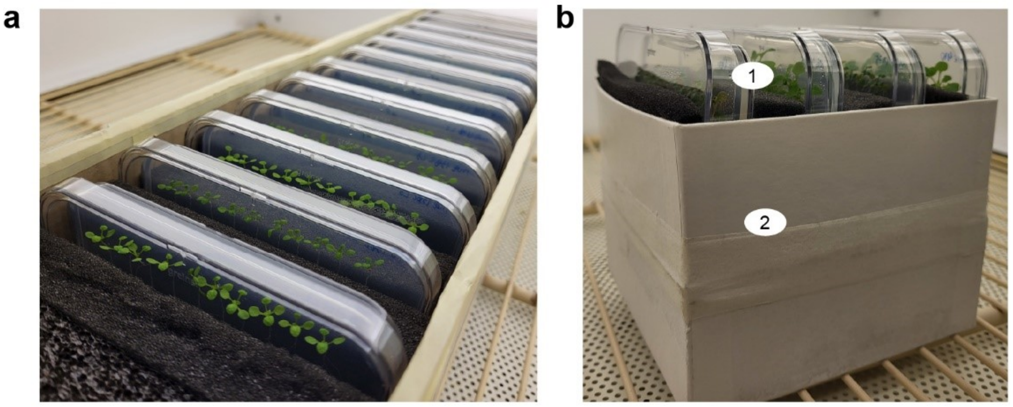

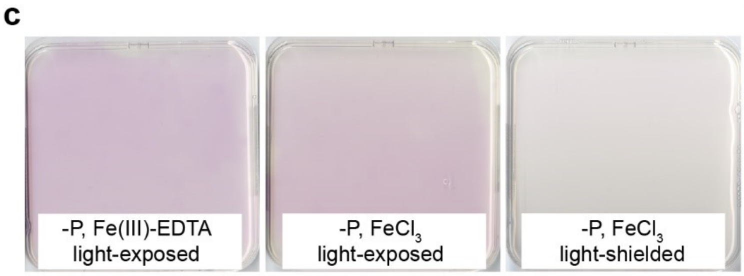

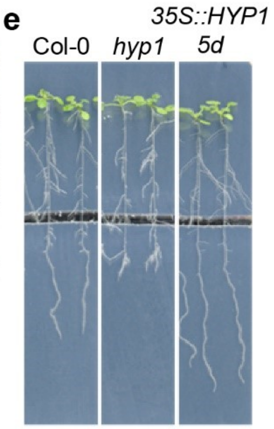

d

Fe source	Fe amount supplied (ng mL^{-1})	
	Calculated	Soluble
100 µM Fe(III)EDTA	4771.65	3171.09 ± 142.83
150 µM $FeCl_3.6H_2O$	8376.59	1.78 ± 0.64

Suppl. Fig. 1. Modified system to grow *A. thaliana* seedlings with roots shielded from direct light exposure.

a-b, Overview of the system. Translucent Petri dishes are placed within a box with dishes separated by a 3-cm thick black foam that extends from the bottom of the box until the height where shoots are located (**a**). At shoot level (**b**, position marked with 1), 130 ± 15 µmol m^{-2} s^{-1} of light reach the plate while at root level (**b**, position marked with 2) 8 ± 5 µmol m^{-2} s^{-1} were detected. **c**, Detection of Fe(II) in the solid agar medium supplied with Fe(III)-EDTA or $FeCl_3$. Translucent Petri dishes placed or not in the light-protected boxes were stained with ferrozine after 3 days inside the growth cabinets used for plant experiments. The appearance of a magenta stain indicates the formation of Fe(II) by photoreduction. **d**, Calculated amount of Fe provided by 100 µM Fe(III)-EDTA (~13% Fe) and 150 µM $FeCl_3$ and determined soluble fraction. The amount of soluble Fe in freshly prepared half-strength MS medium (without agar) containing either 100 µM Fe(III)-EDTA (~13% Fe) or 150 µM $FeCl_3$ were determined by ICP-MS in filtrates recovered after passing the nutrient medium through a 0.45-µm filter. Shown are means ± SD (n = 4 independent measurements). **e**, In a light-protected plate, the short-root phenotype of *hyp1* plants was detected also when potential diffusion of Fe(II) from the light-exposed upper part of the agar was prevented by spatially separating the upper and lower part of the agar at the beginning of the experiment.

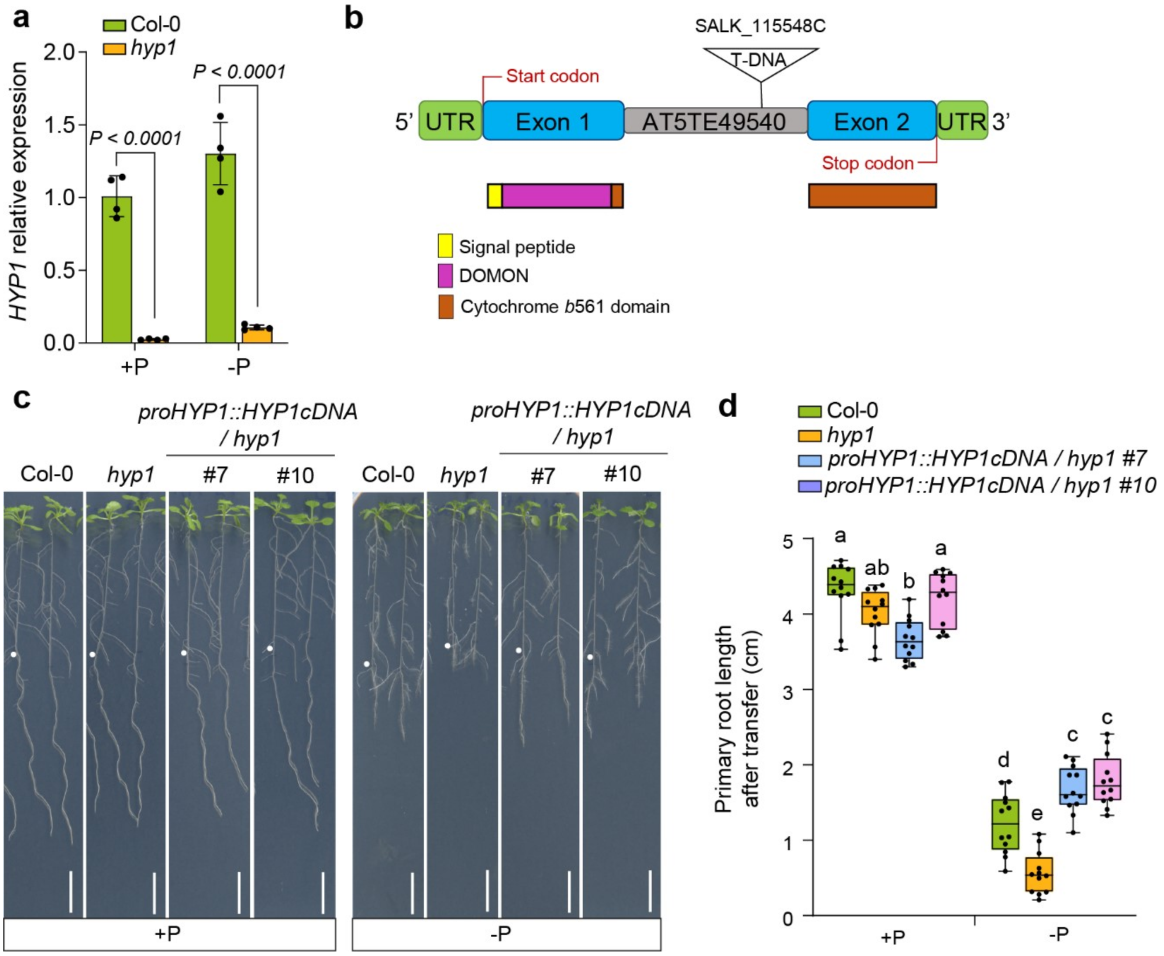

Suppl. Fig. 2. Predicted transposon element in *HYP1* locus is not responsible for the short-root phenotype of *hyp1* insertion mutant.

a, Expression of *HYP1* in roots of wild-type (Col-0) and the insertion mutant *hyp1*. Seven-day-old seedlings were transferred to fresh medium containing 625 µM P (+) or 5 µM P (-P) with 150 µM $FeCl_3$ and whole roots collected for RNA extraction after 4 days. Expression levels were normalized to *UBQ2*. Bars represent means ± SD (*n* = 4 independent root pools). **b**, Exon-intron structure of HYP1, showing the T-DNA insertion in the intro region, which is predicted to encode an *Helitron* transposon element in the forward strand. The approximate position of features of the translated protein relative to genomic DNA is shown. **c-d**, Complementation of primary root hypersensitive of *hyp1* insertion mutant to low P with HYP1 complementary DNA (cDNA). Visual appearance (**c**) and primary root length of wild-type (Col-0), *hyp1* mutant, and two independent lines expressing *proHYP1::HYP1cDNA* in the *hyp1* mutant (*n* = 12 independent roots) (**d**). Ten-day-old seedlings were transferred to fresh medium containing 625 µM P (+) or 5 µM P (-P) with 150 µM $FeCl_3$ and analyzed after 6 days. For the boxplots, central horizontal lines, median; edges of boxes, 25th (bottom) and 75th (top) percentiles; whiskers, minimum and maximum values; and dots, individual biological replicates. Different letters indicate significant differences (one-way ANOVA followed by post-hoc Tukey's test, $P < 0.05$). The exact *P*-values are available in Source Data. In **c**, white dots indicate the position of the primary roots position at the day of transfer. Scale bars, 1 cm (**c**). Source data are provided as a Source Data file.

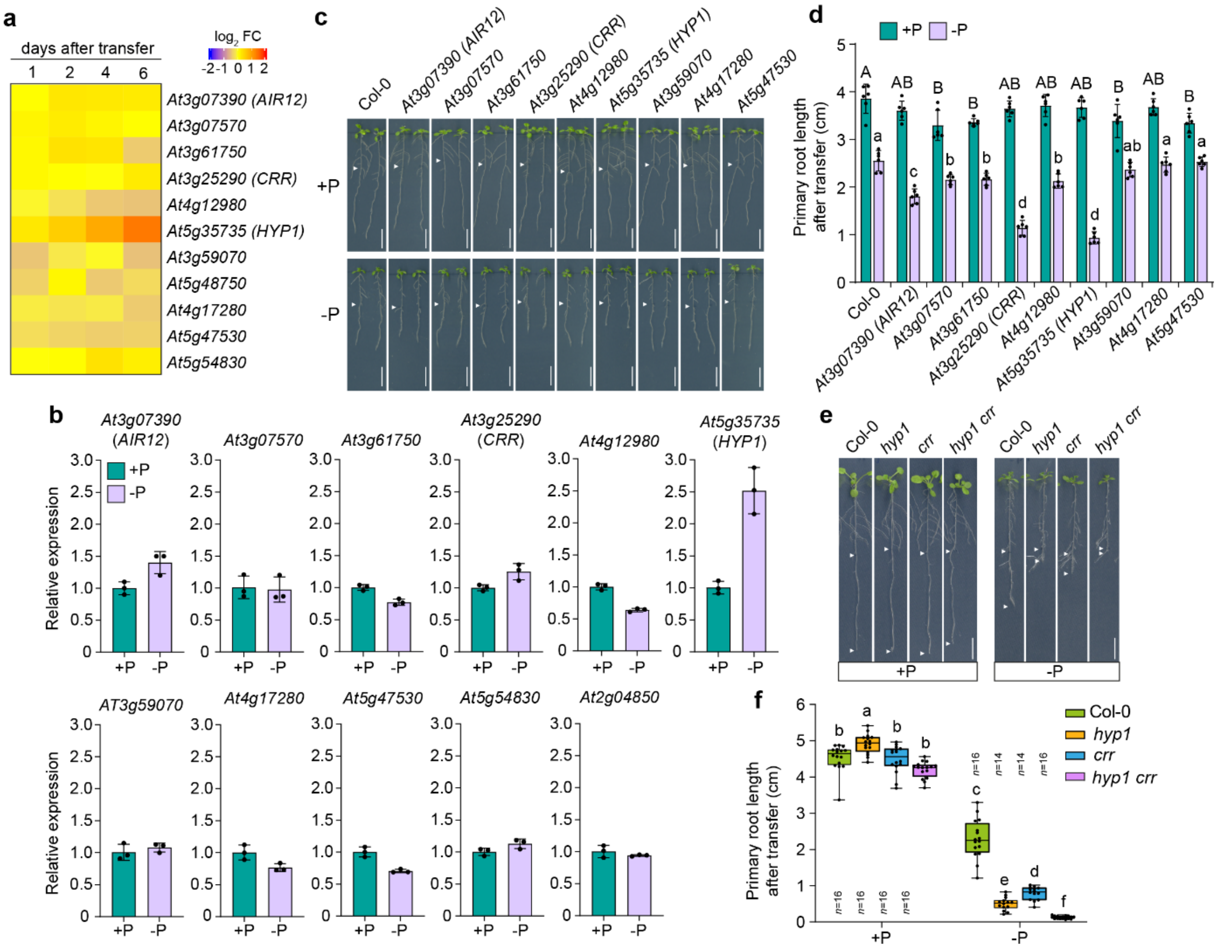

Suppl. Fig. 3. Expression levels of genes encoding DOMON-containing proteins in response to low P and phenotypic analysis of available T-DNA insertion mutants.

a, Heatmap showing the differential expression (|log$_2$ FC| ≥ 1 -P *versus* +P, FDR < 0.05) of *Arabidopsis* genes encoding DOMON-containing proteins during the time course of P treatments according to microarray analysis (*n*= 3 independent root pools). **b**, qPCR validation of the expression of the indicated genes of the DOMON family in response to P deficiency (*n* = 3 independent root pools). Expression levels were normalized to *UBQ2*. Bars represent means ± SD. **c-d**, Primary root sensitivity to low P of available T-DNA insertion mutants for the indicated *CYBDOM* genes. Visual appearance (**c**) and primary root length (*n* = 6 independent roots) (**d**). Seven-day-old seedlings were transferred to fresh medium containing 625 µM P (+) or 5 µM P (-P) with 150 µM $FeCl_3$ and analyzed after 6 days. White arrows indicate the position of the primary roots at the day of transfer. Bars represent means ± SD. **e-f**, Phenotypic characterization of a *hyp1 crr* double mutant. Visual appearance (**e**) and primary root length (*n* = independent roots as indicated) of wild-type (Col-0), *hyp1*, *crr* and *hyp1 crr* plants (**f**). Seven-day-old seedlings were transferred to fresh medium containing 625 µM P (+) or 5 µM P (-P) with 150 µM $FeCl_3$ and analyzed after 6 days. Upper white arrows indicate the position of the primary roots at the day of transfer and the bottom arrow the position of the primary root 6 days later. For the boxplots, central horizontal lines, median; edges of boxes, 25th (bottom) and 75th (top) percentiles; whiskers, minimum and maximum values; and dots, individual biological replicates. In **d** and **f**, different letters indicate significant differences (one-way ANOVA followed by post-hoc Tukey's test, $P < 0.05$). The exact *P*-values are available in Source Data. Scale bars, 1 cm (**c**). Source data are provided as a Source Data file.

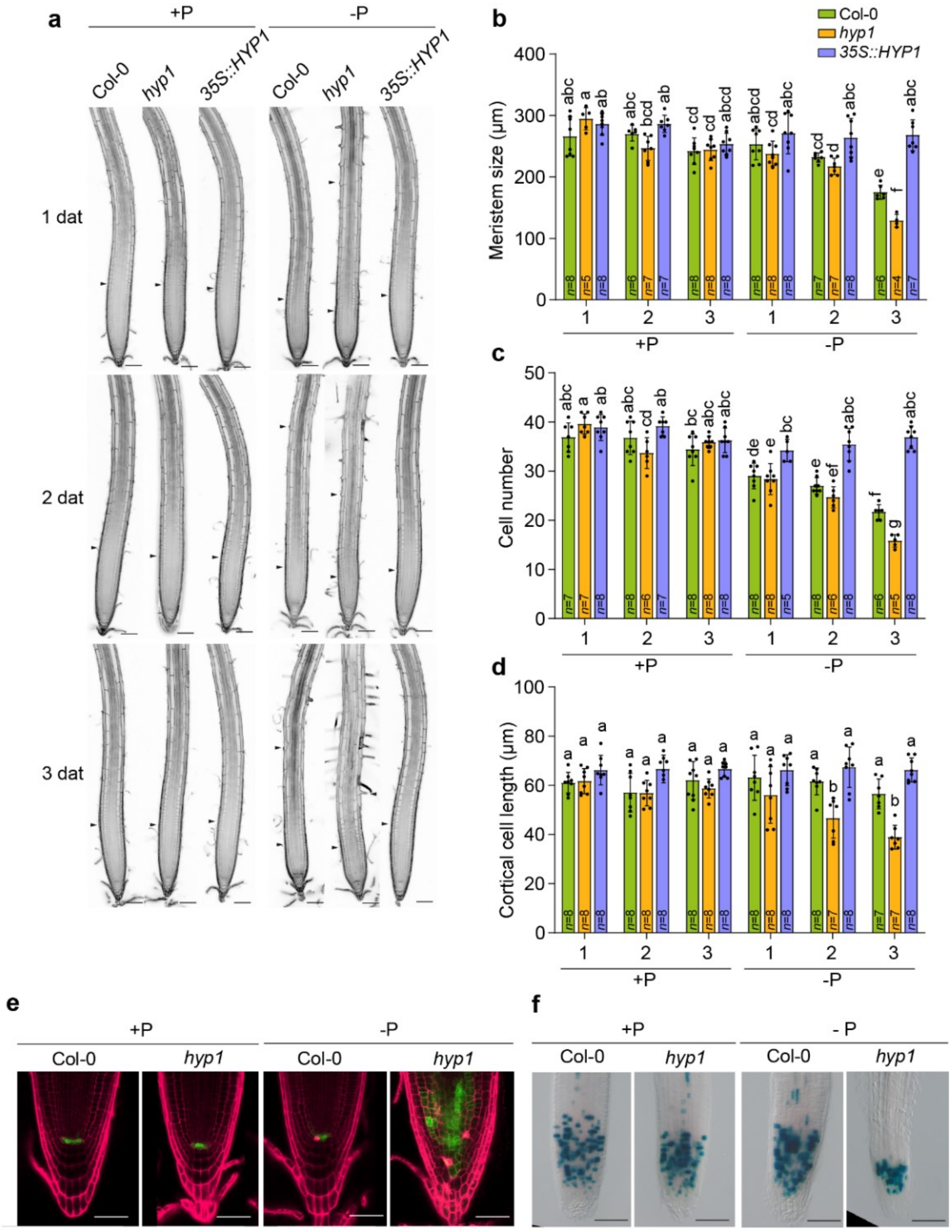

Suppl. Fig. 4. HYP1 prevents inhibition of cell elongation and loss of meristematic integrity under low P conditions.

a-d, Time-dependent changes in root tip integrity and mature cell length after transferring seven-day-old seedlings to fresh medium containing 625 µM P (+P) or 5 µM P (-P) with 150 µM $FeCl_3$. Confocal images of propidium iodide-stained root tips (**a**) and quantification of meristem cell length (**b**), meristem cell number (**c**) and mature cortical cell length (**d**). Bars represent means ± SD (*n* = independent roots as indicated). All individual data points are plotted. The letters indicate statistical significance (one-way ANOVA followed by post-hoc Tukey's test, $P < 0.05$). The exact *P*-values are available in Source Data. In **a**, arrowheads indicate the boundary between meristem and transition zone. **e-f**, Expression of the quiescent center marker *proWOX5::GFP* (**e**) and of the mitotic activity reporter *proCYCB1;1::GUS* (**f**) in root tips of wild-type (Col-0) and *hyp1* plants. Seven-day-old seedlings were transferred to fresh medium containing 625 µM P (+P) or 5 µM P (-P) with 150 µM $FeCl_3$ and analyzed 6 days after transfer (dat). The experiment was repeated two times with similar results and representative images from one experiment are shown. Scale bars, 100 µm (**a** and **f**), and 50 µm (**e**). Source data are provided as a Source Data file.

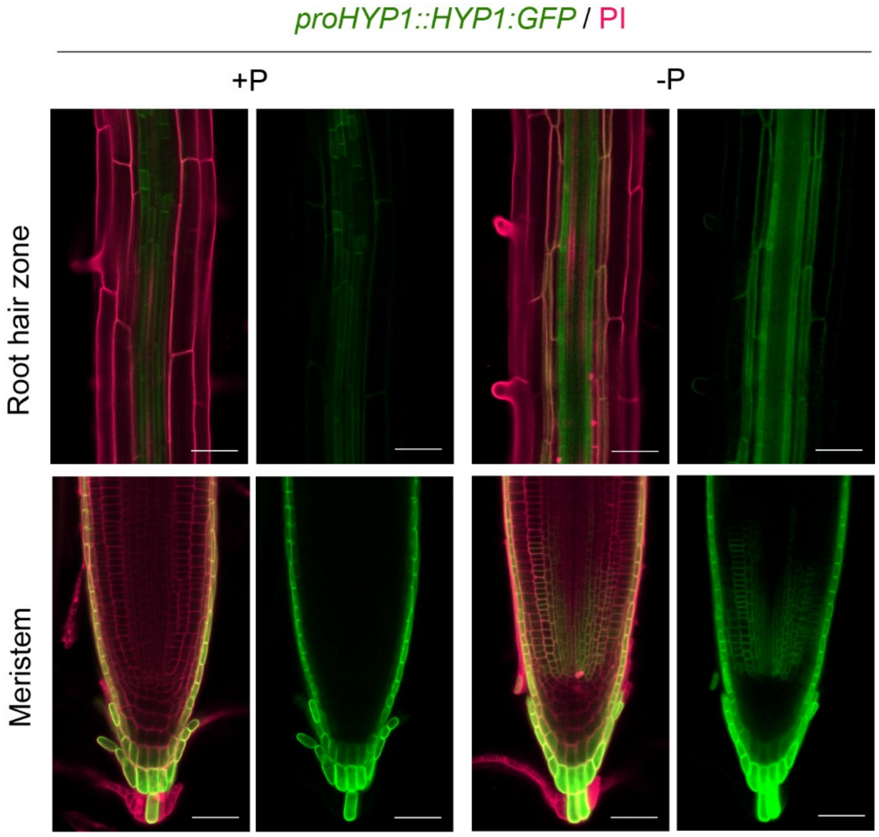

Suppl. Fig. 5. Cell type-specific localization of HYP1 in different root zones.
Localization of HYP1:GFP in the apical root meristem of *A. thaliana* plants. Seven-day-old seedlings were transferred to fresh medium containing 625 µM P (+P) or 5 µM P (-P) with 150 µM $FeCl_3$. HYP1:GFP localization in the meristem and root hair zone of roots counterstained with the cell wall stain propidium iodide (PI) after 3 days on the indicated treatments. The experiment was repeated three times with similar results and representative images from one experiment are shown. Scale bars, 50 µm.

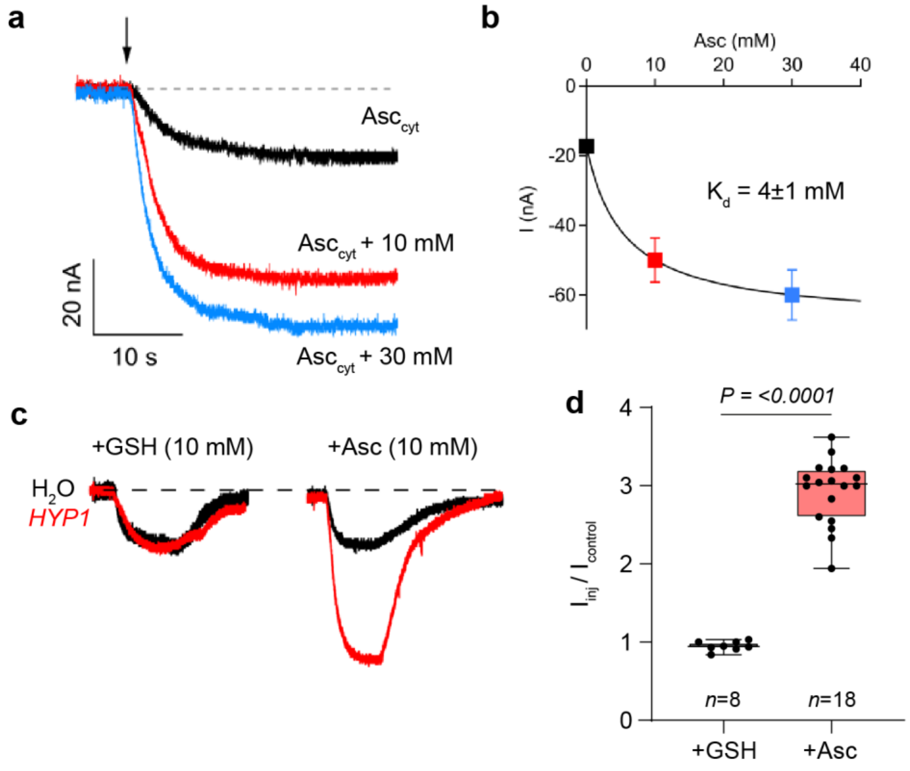

Suppl. Fig. 6. HYP1-mediated currents in *X. laevis* oocytes depend on cytosolic ascorbate.
a, Membrane currents elicited by 1 mM $[Fe(CN)_6]^{3-}$ (ferricyanide) in oocytes injected with *HYP1* cRNA before ascorbate injection (Asc_{cyt}, black trace), after a first ascorbate injection corresponding to Asc_{cyt} + 10 mM (red trace) and after a second ascorbate injection corresponding to Asc_{cyt} + 30 mM (blue trace). Holding potential at -20 mV. **b**, Dependence of *HYP1*-elicited currents on the ascorbate concentration resulting from ascorbate injection (Asc_{inj}). Data points were subjected to a fit with the Michaelis-Menten function $I=I_{max}/(1+K_D/(Asc_{cyt} + Asc_{inj})$ (continuous lines) giving I_{max} = 68 ± 8 nA, K_D=4 ± 1 mM, Asc_{cyt} = 1.5 ± 0.2 mM. Data represent mean ± SD (*n*= 6 different oocytes). **c**, HYP1-mediated currents elicited by 1 mM $[Fe(CN)_6]^{3-}$ (ferricyanide) before (black traces) and after (red traces) injection with the indicated concentrations of glutathione (GSH) and ascorbate (Asc). **d**, Ratio of currents before ($I_{control}$) and after oocyte injection (I_{inj}) respectively with glutathione (+GSH) and ascorbate (+Asc), calculated from results obtained in experiments as shown in **c**. For the boxplots, central horizontal lines, median; edges of boxes, 25th (bottom) and 75th (top) percentiles; whiskers, minimum and maximum values; and dots, individual biological replicates (*n* = independent oocytes as indicated). *P* value according to two-sided, Student's *t*-test. Source data are provided as a Source Data file.

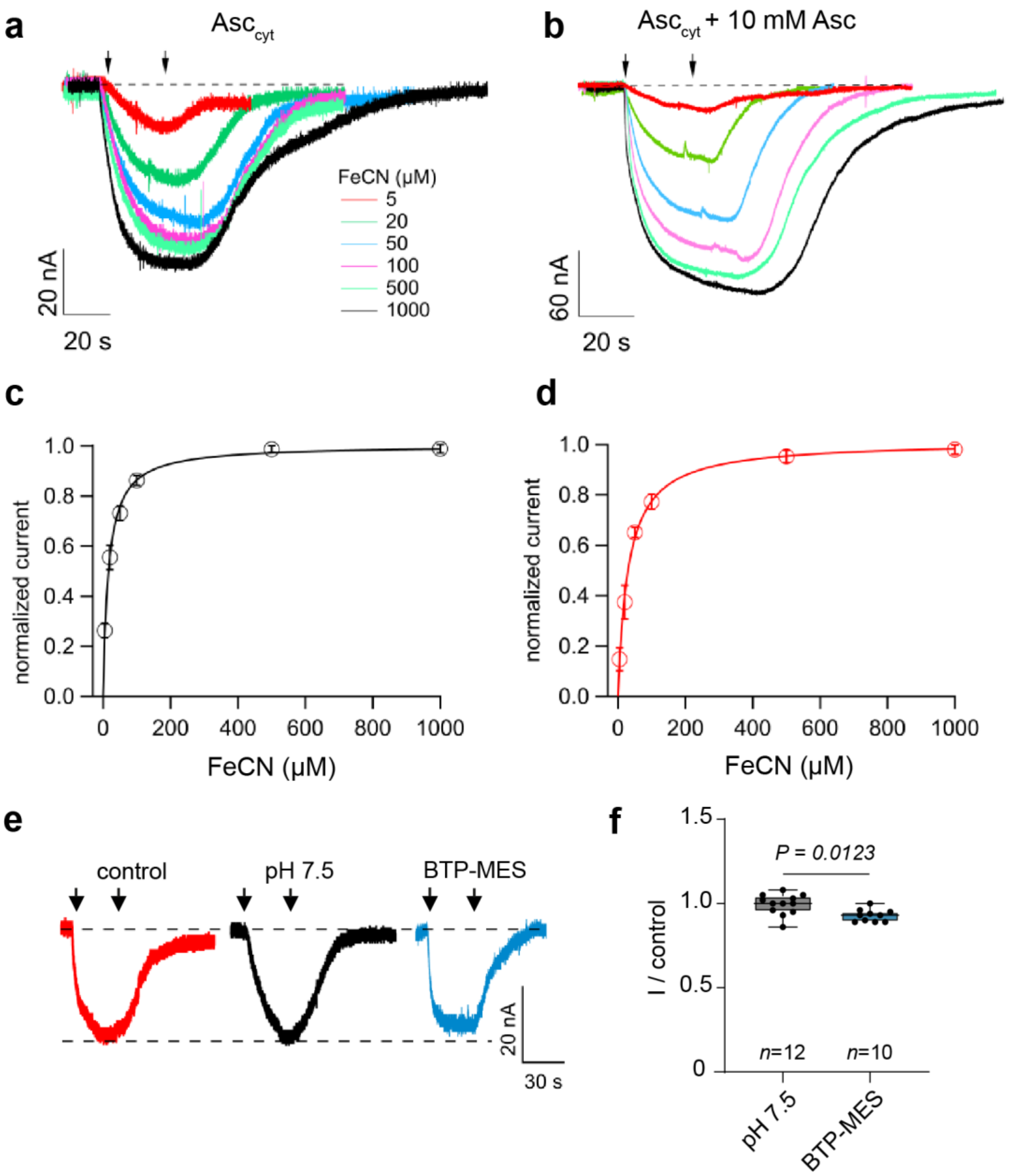

Suppl. Fig. 7. HYP1-mediated electron currents in *X. laevis* oocytes in dependence of external concentrations of electron acceptor.

a-b, Membrane currents elicited by increasing external $[Fe(CN)_6]^{3-}$ (ferricyanide, indicated by the arrows) concentrations in oocytes injected with *HYP1* cRNA before ascorbate injection (**a**) and after ascorbate injection (**b**). Holding voltage at -20 mV. Note the difference in the current scales. **c-d**, Dependence of HYP1-elicited currents on external $[Fe(CN)_6]^{3-}$ (ferricyanide; FeCN) before (**c**) and after ascorbate injection (**d**). Data points were normalized to the value at 1 mM FeCN/Asc$_{cyt}$ and fitted with a Michaelis–Menten function (continuous lines) giving the following parameters: K_F(Asc$_{cyt}$) = 18 ± 2 µM, K_F(Asc$_{cyt}$ + 10 mM) = 30 ± 2 µM. Symbols represent means ± SD (*n* = 10 independent oocytes in Asc$_{cyt}$ and *n* = 6 independent oocytes in Asc$_{cyt}$ + 10 mM Asc). **e-f**, Membrane current recordings (**e**) and normalized currents to control (**f**) in oocytes injected with *HYP1* cRNA upon exposure to 1 mM $[Fe(CN)_6]^{3-}$ (ferricyanide; FeCN) in control solution (red trace) or in modified solutions with pH set to 7.5 (black trace) and with with NaCl, KCl, $CaCl_2$ and $MgCl_2$ replaced with BTP-MES (blue trace). Holding voltage at -20 mV. In **a**, **b** and **c**, the left and right arrows indicate the addition and removal of ferricyanide, respectively. For the boxplots in **f**, central horizontal lines, median; edges of boxes, 25th (bottom) and 75th (top) percentiles; whiskers, minimum and maximum values; and dots, individual biological replicates (*n* = independent oocytes as indicated). *P* value according to two-sided, Student's *t*-test. Source data are provided as a Source Data file.

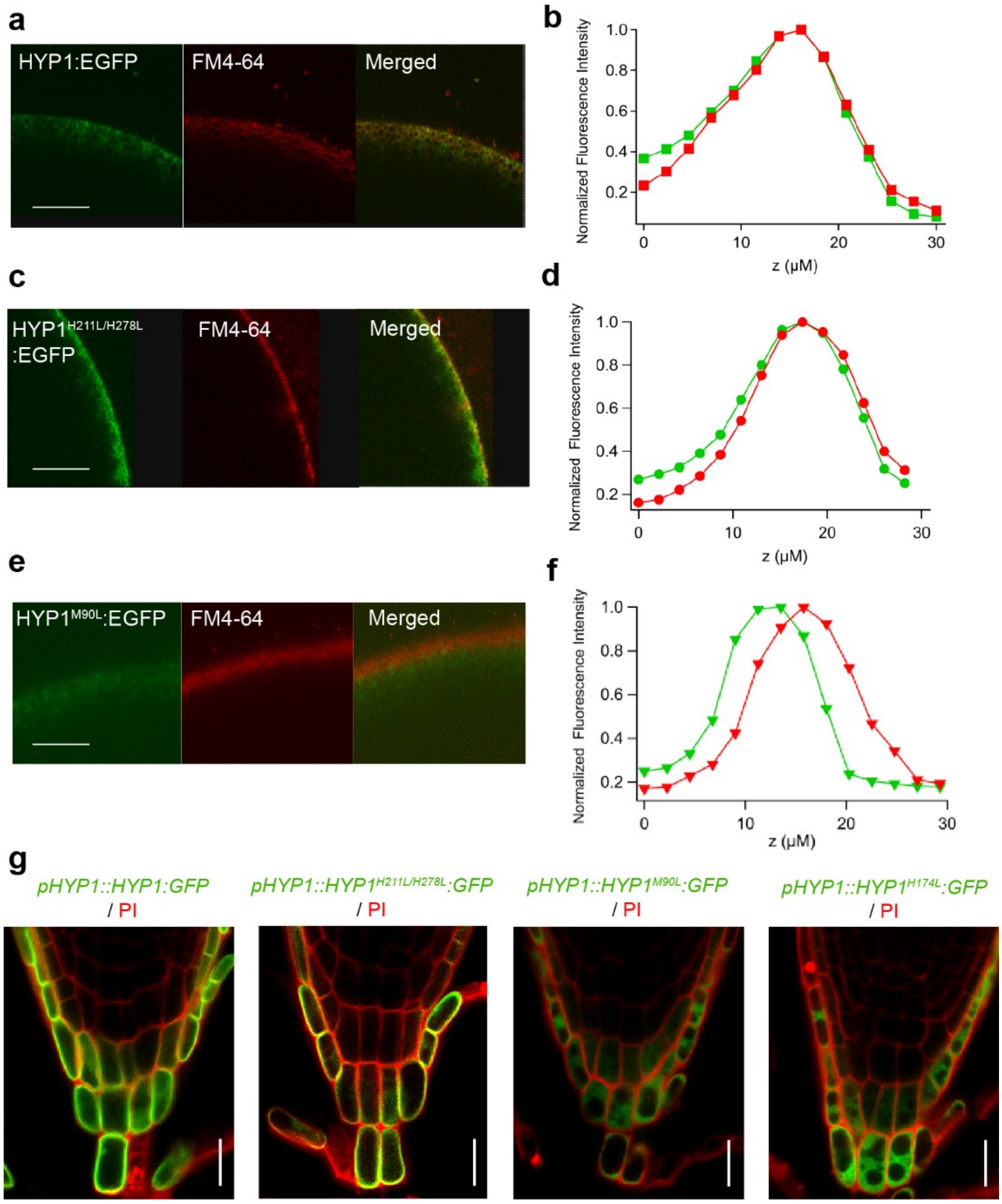

Suppl. Fig. 8. Plasma membrane localization of wild-type and mutated HYP1 variants.
a-f, HYP1 localization in oocytes. Confocal images showing the border of an oocyte expressing wild type *HYP1:EGFP* (**a**) or the *HYP1*$^{H211L/H278L}$*:EGFP* (**c**) and *HYP1*^{M90L}*:EGFP* (**e**) mutated variants. Plasma membrane was stained with FM4-64. The fluorescence intensity profiles of *EGFP* (green) and FM4-64 signals (red), calculated along the z axis of the oocyte border at the bottom for wild type *HYP1:EGFP* (**b**) or the *HYP1*$^{H211L/H278L}$*:EGFP* (**d**) and *HYP1*^{M90L}*:EGFP* (**f**) mutated variants. The fluorescence was normalized at the maximal value for each signal. Similar results were obtained in *n* = 6 independent oocytes expressing *HYP1:EGFP*, *n* = 6 with *HYP1*$^{H211L/H278L}$*:EGFP* and *n* = 4 with *HYP1*^{M90L}*:EGFP*. **g**, Localization of wild type HYP1:GFP and three variants carrying mutations in amino acid residues putatively involved with *b*-heme coordination in the DOMON domain. The constructs were expressed in the *hyp1* background. Localization was assessed in roots counterstained with the cell wall stain propidium iodide (PI) of plants grown under low P for 3 days. The experiment was repeated three times with similar results and representative images from one experiment are shown. Scales, 30 μm (**a**, **c, e**) and 20 μm (**g**).

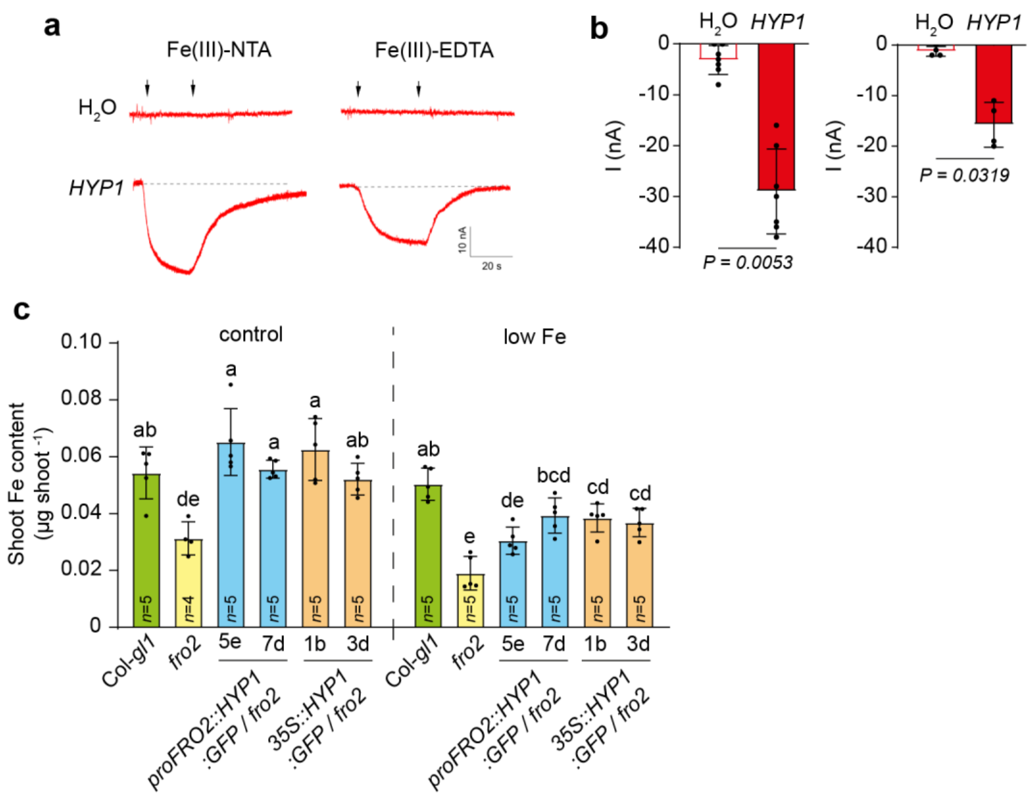

Suppl. Fig. 9. HYP1 mediates electron transfer to ferric-chelates in oocytes and affects shoot Fe accumulation *in planta*.

a-b, HYP1-mediated currents (**a**) and mean amplitudes [Fe(III)-NTA, *n* = 7 independent oocytes for H_2O and *n* = 7 for HYP1; Fe(III)-EDTA, *n* = 4 independent oocytes for H_2O and *n* = 4 HYP1]. (**b**) in response to external availability of 1 mM of Fe(III)-nitrilotriacetate [Fe(III)-NTA] or Fe(III)-Ethylenediaminetetraacetic acid [Fe(III)-EDTA]. Oocytes were injected with water (H_2O) or *HYP1* cRNA (*HYP1*) and recordings made after raising cytosolic concentration to 10 mM ascorbate. The left and right arrows indicate the addition and removal of the ferric chelate, respectively. Holding voltage of -20 mV. Bars represent means ± SD *P* values according to two-sided, Student's *t*-test. **c**, Shoot Fe contents of wild-type (Col-*gl1*), *fro2*, and *fro2* plants expressing *HYP1:GFP* under the control of *FRO2* or CaMV *35S* promoter grown for 6 days on 75 µM (control) or 25 µM Fe-EDTA (low Fe). Bars represent means ± SD (*n* = independent pools with 6 shoots each, as indicated in the plots). Different letters indicate significant differences (one-way ANOVA followed by post-hoc Tukey's test, $P < 0.05$). The exact *P*-values are available in Source Data. Source data are provided as a Source Data file.

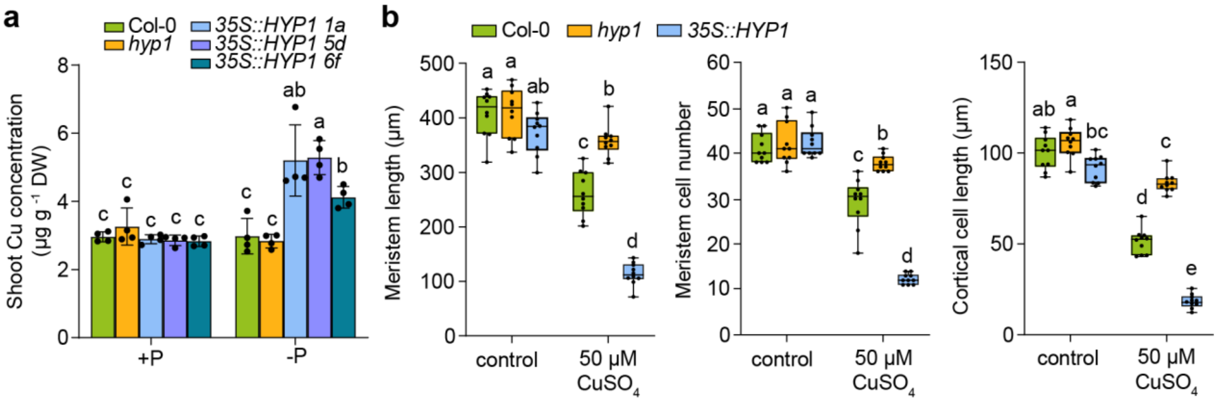

Suppl. Fig. 10. *HYP1* overexpression stimulates shoot Cu accumulation under low P and decreases root meristem size and cell elongation under high Cu supply.
a, Shoot Cu concentration of wild-type (Col-0), *hyp1* mutant, and three independent transgenic lines expressing *35S::HYP1* in Col-0 after long-term growth on low-P conditions. Ten-day-old seedlings were transferred to fresh medium containing 625 µM P (+P) or 5 µM P (-P) and analyzed after 20 days. Bars represent means ± SD ($n = 4$ independent pools containing 4 plants each). **b**, Changes in meristem cell length, meristem cell number, and mature cortical cell length in primary roots of wild-type (Col-0), *hyp1* mutant and one *HYP1*-overexpressing line in response to high Cu concentrations (n = 10 independent roots). For the boxplots, horizontal line, median; edges of boxes, 25th (bottom) and 75th (top) percentiles; whiskers, minimum and maximum values; and dots, individual biological replicates. In **a** and **b**, different letters indicate significant differences (one-way ANOVA followed by post-hoc Tukey's test, $P = 0.05$). The exact *P*-values are available in Source Data. DW, dry weight. Source data are provided as a Source Data file.

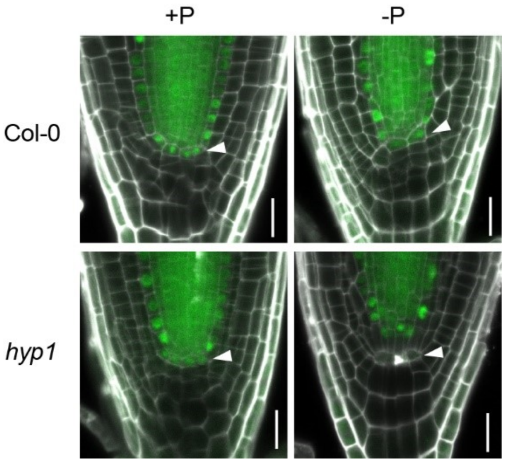

Suppl. Fig. 11. Loss of HYP1 impairs SHR movement in root meristems under low P.
Confocal images of root tips of wild-type (Col-0) and *hyp1* plants expressing *proSHR::SHR:GFP*. Seven-day-old seedlings were transferred to fresh medium containing 625 µM P (+P) or 5 µM P (-P). SHR:GFP protein localization in the root apical meristem after 3 days on the indicated treatments. Cell walls were stained with propidium iodide (white fluorescence) to reveal the cell borders. The experiment was repeated two times with similar results and representative images from one experiment are shown. Arrowheads indicate the position of the quiescent center. Scale bars, 20 µm.

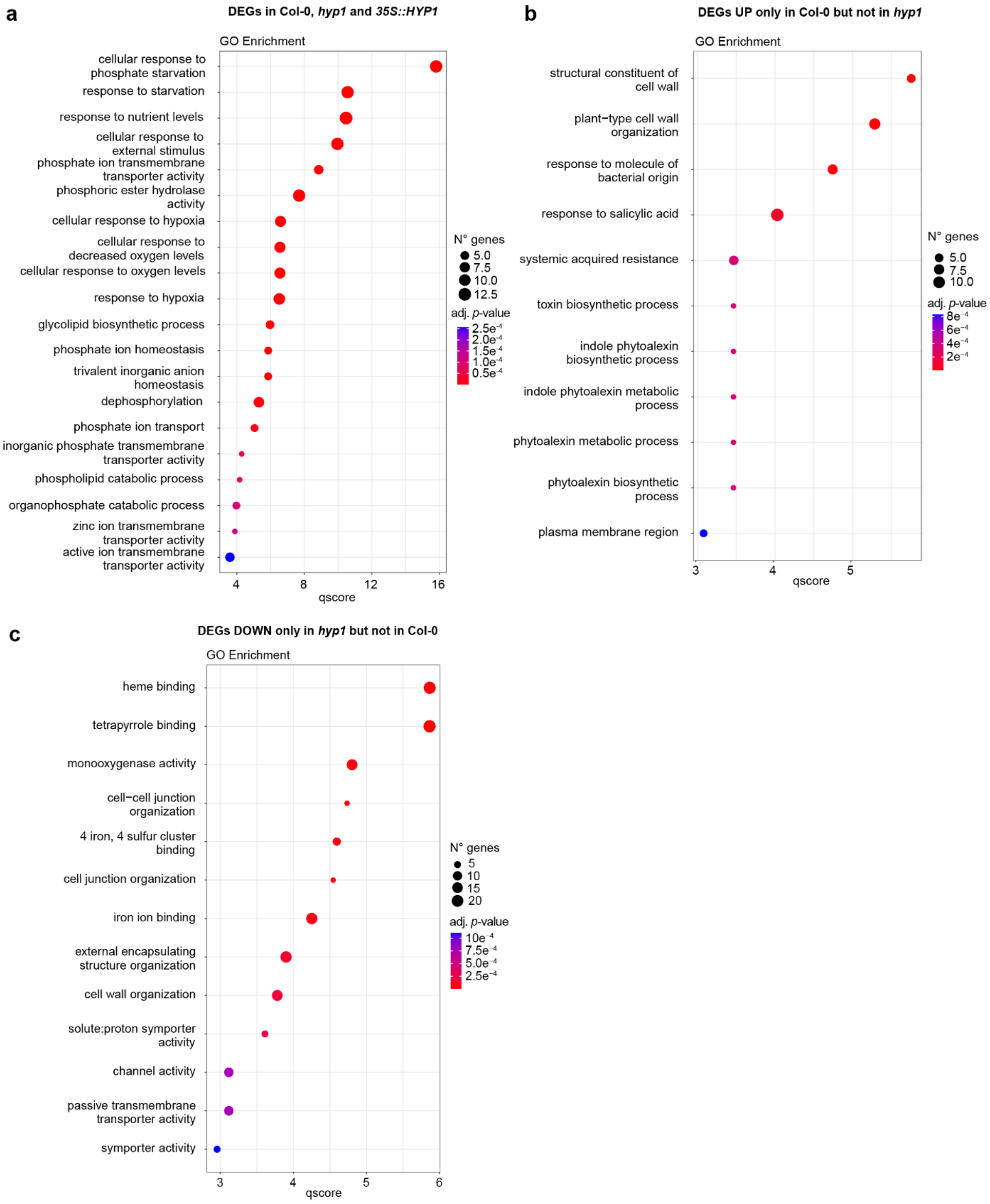

Suppl. Fig. 12. Differential response of the transcriptome in lines with modulated HYP1 expression.

a-c, Most significant GO terms among differentially expressed genes in response to low P in root tips (|log2 FC| ≥ 1 -P *versus* +P, *FDR* < 0.05) of wild-type (Col-0), *hyp1* and *35S::HYP1* plants (**a**), only significantly up-regulated in roots of Col-0 but not in *hyp1* (**b**), or only significantly down-regulated in roots of *hyp1* but not in Col-0 (**c**). Ten-day-old seedlings were transferred to fresh medium containing 625 µM P (+P) or 5 µM P (-P) with 150 µM $FeCl_3$ and root tips (5 mm from apex) were collected for whole transcriptome sequencing (RNA-seq) after 3 days. Each treatment consisted of three independent biological replicates (*n* = 3 independent root pools). *P*-values are adjusted Benjamini-Hochberg FDR.

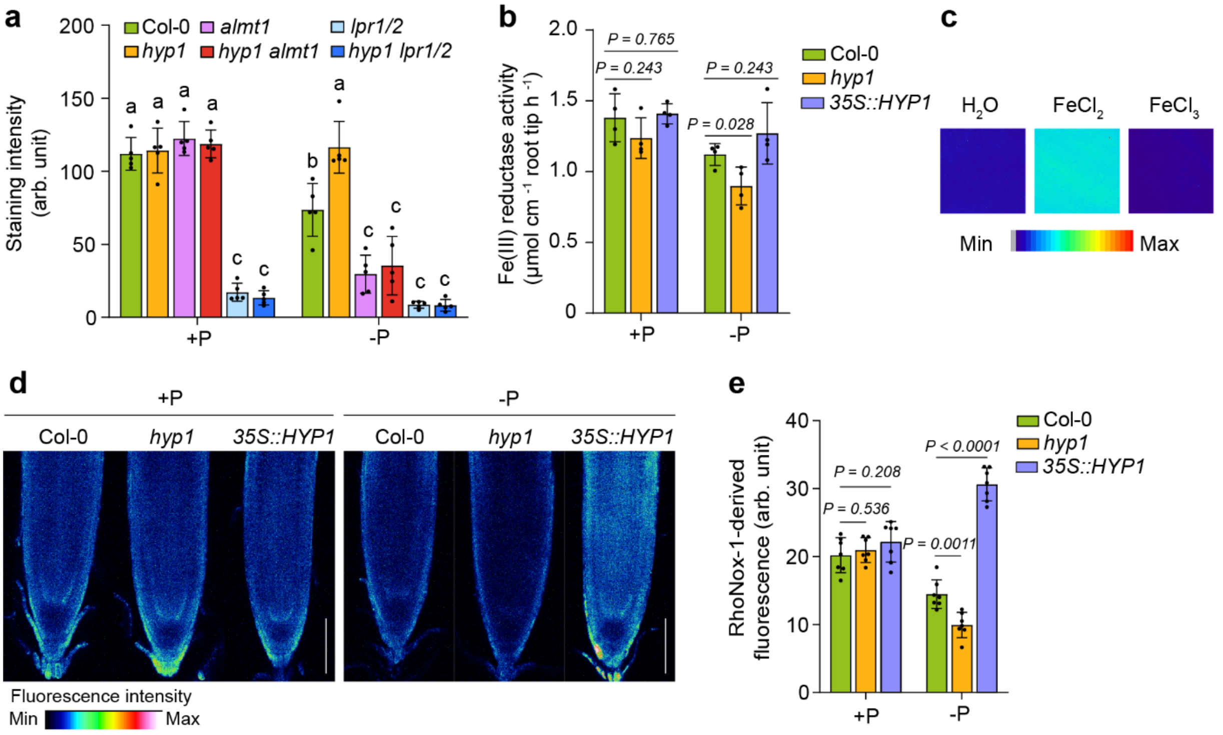

Suppl. Fig. 13. HYP1-dependent ferric reduction in root tips of P-deficient plants.
a, Quantification of Perls/DAB shown in Fig. 6c. Bars represent means ± SD (*n* = 5 independent roots). **b**, Ferric-chelate reductase activity of detached root tips of wild-type (Col-0), *hyp1* and *35S::HYP1* plants. Ten-day-old seedlings were transferred to fresh medium containing 625 µM P (+P) or 5 µM P (-P) with 150 µM $FeCl_3$ and assayed after 1 days using Fe(III)-malate as substrate. Bars represent means ± SD (*n* = 4 replicates consisting of 6 root tips each). *P* values (two-sided, Student's *t*-test). **c-d**, Reaction of the Fe(II)-sensitive fluorescent dye RhoNox-1 to solutions containing $FeCl_2$ and $FeCl_3$ (**c**), representative images (**d**) and quantification of RhoNox-1-derived signals (**e**) in root tips of wild-type (Col-0), *hyp1* and *35S::HYP1* plants (*n* = 7 independent roots). Ten-day-old seedlings were transferred to fresh medium containing 625 µM P (+) or 5 µM P (-P) and stained with RhoNox-1 after 1 day. In **a**, **b**, and **e**, bars represent means ± SD and different letters indicate significant differences (one-way ANOVA followed by post-hoc Tukey's test, *P* < 0.05). The exact *P*-values are available in Source Data. Scale bars, 100 µm. Source data are provided as a Source Data file.

Supplementary Table 1. Primers for genotyping T-DNA insertion lines.

T-DNA line	Primer sequence
SALK_115548 (*hyp1*)	For: ACAATGAACCAAGTGAGTGGC
	Rev: TTACTAAATCATTTGGAACCCAC
SALK_202530 (*crr-1*)	For: GCCAAAAGCCTTTACGGTTAC
	Rev: CTTGTGATCTTTCTTAGGCCG
SALK_009629 (*almt1*)	For: GAAATTATTTGGGGAAGCTGC
	Rev: TCTTTACCCATGGGAAAAACC
SALK_016297 (*lpr1*)	For: CTCATCGCCAGTAGGTAGCTG
	Rev: ACTCATGGGTGTGAACCAAAG
SALK_091930 (*lpr2*)	For: CATAGCCTGGCTCTTGAGTTG
	Rev: GTCATAGCTCAGTCGAATCGC

Supplementary Table 2. Primers used for real-time quantitative PCR.

Fragment	Primer sequence
qPCR At2g36170 (*UBIQUITIN 2*)	For: AGACGAACGCAAAGATGCAG
	Rev: CCGGCGAAGATCAACCTCTG
qPCR At2g04850	For: TGATGGGCGTGGTGAATGTG
	Rev: ACCAGCGTCGACAGACATAG
qPCR At3g07390 *(AIR12)*	For: CTCCCACCGTGTGTTGAGTT
	Rev: GTTCCTCGTCAATGACCCCG
qPCR At3g07570	For: CCCCACTTGGTTCTATGCCC
	Rev: ATTATTGGCCTTGAGCCGGT
qPCR At3g61750	For: CGAATGGCGGATAATGGAGGA
	Rev: AGATGGTGAGCCGATAGTGC
qPCR At3g25290 *(CRR)*	For: ACCATGGAGTCGGTTATGCG
	Rev: AGAGCAAAGCAATGCCTCCA
qPCR At4g12980	For: ACCACCACGGAGTTGGATAC
	Rev: CGTGATGCCTCCTAAGGTCC
qPCR At5g35735 *(HYP1)*	For: CGAGCCACTCACTTGGTTCA
	Rev: CTAGGCGTCCTGGTGATGTG
qPCR At3g59070	For: ATCGGAGAGACGGAAAAGGC
	Rev: AGAAGCGGCGGAGAAGTATG
qPCR At4g17280	For: GAGTCCCAAGAAGCAGTGGA
	Rev: TAGATGCTCCATGCTGAGCC
qPCR At5g47530	For: GAGAAACGCTTACACCGCCA
	Rev: ACCATCGTTGCCTACTCGTT
qPCR At5g54830	For: ACGTGGACGGGCTTAACTTG
	Rev: TCCACATTCCCCAAAACCCAA
qPCR At5g48750	For: TGCACGCCATGAGTGGAAAT
	Rev: TCCCCCAACACACTGCATTC

Supplementary Table 3. Primers used for cloning and site-directed mutagenesis.

Fragment	Primer sequence
HYP1 promoter for pENTR	For: CACCTGGTGAAGCGTTGACTCTC
	Rev: GTCTTCGTGTTCTGTTTTGAAAG
*Bsa*I mutation in *HYP1* promoter	For: GGTGGGACTCAAACTCTCAATGG
	Rev: GTTTGAGTCCCACCCAAAAAAAGC
HYP1 promoter for pGGA000	For: AACAGGTCTCAACCTTGGTGAAGCGTTGACTCTC
	Rev: AACAGGTCTCATGTTGTCTTCGTGTTCTGTTTTGAAAG
HYP1 ORF for pGGC000	For: AACAGGTCTCAGGCTAAATGGACCGAACACAATCTC
	Rev: AACAGGTCTCACTGAGGCGTCCTGGTGATGTGG
FRO2 promoter for pENTR	For: CACCGCGTTTTCTTGTTACCAATCG
	Rev: CCTCTCTTTCCTCTCAGGATTTC
*Bsa*I mutation in *FRO2* promoter	For: GGAGGACTCATATCTTCTTCAACCC
	Rev: ATATGAGTCCTCCCAAATTGGAT
FRO2 promoter for pGGA000	For: AACAGGTCTCAACCTGCGTTTTCTTGTTACCAATCG
	Rev: AACAGGTCTCATGTTCCTCTCTTTCCTCTCAGGATTTC
HYP1^{M90L} mutation	For: TCAGCTGGTTGGGACGCAAG
	Rev: CAACCAGCTGAGTACTACTTGGG
HYP1^{H174L} mutation	For: CGAGTCTTCAAACCTCAGGAGATAATATG
	Rev: GTTTGAAGACTCGCAGGAACACC
HYP1^{H211L} mutation	For: AGACGCTTGGAGTACTAAATGC
	Rev: CTCCAAGCGTCTGTTTTCATAAAG
HYP1^{H278L} mutation	For: AACCCTTCGTAACCTTGGAATAG
	Rev: TACGAAGGGTTGAGTAAGATG
HYP1 cDNA in pGemHE	For: TAAGCACCCGGGATGGACCGAACACAATCTC
	Rev: TGCTTATCTAGACTAGGCGTCCTGGTGATGTGG
BRN1 promoter for pGGA000	For: AACAGGTCTCAACCTGGTCAATTCTGGTTTCCGTTA
	Rev: ACCAGGTCTCATGTTTCGGTTATTAAATGATCAATGTTT
SGN1 promoter for pGGA000	For: AACAGGTCTCAACCTATGAGGACCCTGTGCATTG
	Rev: AACAGGTCTCATGTTTTTTTGTGTTGTAAGATTTTAGGAG
LPR1 promoter for pGGA000	For: AACAGGTCTCAACCTCCTAGAATGTTATTGATGTTTCTTG
	Rev: AACAGGTCTCATGTTTTCTGACAAGTCAATTCAGTTTTGA

Supplementary Table 4. Protocol for fixation, dehydration and embedding of root tissue.

Combined conventional & microwave assisted root tissue preparation in a PELCO Bio Wave®34700-230 (Ted Pella, Inc., Redding CA, USA)				
Process	Reagent	Power [W]	Time [sec]	Vacuum [mm Hg]
1. Fixation	2.0% (v/v) glutaraldehyde and 2.0% (v/v) paraformaldehyde in 0.05 M cacodylate buffer (pH 7.2)	150<br>0<br>150<br>0<br>150	60<br>60<br>60<br>60<br>60	0<br>0<br>0<br>0<br>0
	After step samples were kept for additional overnight on a shaket at 4 °C			
2. Wash	1x 0.05 M cacodylate buffer (pH 7.2) and 3x distilled water	150	60	0
5. Dehydration	Ethanol series: 30%, 40%, 50%, 60%, 70%, 80%, 90%, 2x 100%, 100% acetone	150	60	0
	After each step samples were kept for additional 5 min on a shaker at RT			
6. Resin infiltration	25% Spurr resin in acetone<br>50% Spurr resin in acetone<br>75% Spurr resin in acetone<br>100% Spurr resin in acetone	2 hrs on shaker at RT<br>2 hrs on shaker at RT<br>2 hrs on shaker at RT<br>overnight on shaker at RT		
5. Polymerization	24 hs at 100 °C in propolymerized flat embedding moulds with disc in a heating cabinet at 70 °C			
