# Supplementary material for: Ferric reduction by a CYBDOM protein counteracts increased iron availability in root meristems induced by phosphorus deficiency
Source: Nat Commun. 2024 Jan 11;15:422. doi: 10.1038/s41467-023-43912-w (PMC10784544; doi:10.1038/s41467-023-43912-w)
Supplement: Supplementary file 3 — Description of Additional Supplementary Files [file 41467_2023_43912_MOESM3_ESM.pdf]

Description of Additional Supplementary Files

Supplementary Data 1. Genes significantly regulated in response to P deficiency in roots of Arabidopsis thaliana (Col-0) after 1, 2, 4 or 6 days. Seven-day-old seedlings were transferred to fresh medium containing 625 µM P (+P) or 5 µM P (-P) with 100 µM Fe(III)-EDTA.

Supplementary Data 2. Significantly enriched GO terms of cluster 1.

Supplementary Data 3. Expression of selected genes in roots of Arabidopsis thaliana (Col-0) after transfer for 1, 2, 4 or 6 days to low P. Seven-day-old seedlings were transferred to fresh medium containing 625 µM P (+P) or 5 µM P (-P) with 100 µM Fe(III)-EDTA.

Supplementary Data 4. Genes significantly regulated in response to P deficiency in root tips of wild-type (WT; Col-0), hyp1 mutant and one HYP1-overexpressing line (HYP1OX). Ten-day-old seedlings were transferred to fresh solid medium containingto 625 µM P (+P) or 5 µM P (-P) with 150 µM FeCl3 and root samples collected for RNA-seq 3 days after transfer.

Supplementary Data 5. Expression of selected genes in response to low P in root tips of wild-type (WT; Col-0), hyp1 mutant and one HYP1-overexpressing line (HYP1OX). Ten-day-old seedlings were transferred to fresh solid medium containingto 625 µM P (+P) or 5 µM P (-P) with 150 µM FeCl3 and root samples collected for RNA-seq 3 days after transfer.
