# Supplementary material for: Ferric reduction by a CYBDOM protein counteracts increased iron availability in root meristems induced by phosphorus deficiency
Source: Nat Commun. 2024 Jan 11;15:422. doi: 10.1038/s41467-023-43912-w (PMC10784544; doi:10.1038/s41467-023-43912-w)
Supplement: Supplementary file 9 — Reporting Summary [file 41467_2023_43912_MOESM9_ESM.pdf]

nature portfolio

Corresponding author(s): Ricardo F. H. Giehl
Last updated by author(s): Nov 17, 2023

Reporting Summary

Nature Portfolio wishes to improve the reproducibility of the work that we publish. This form provides structure for consistency and transparency in reporting. For further information on Nature Portfolio policies, see our Editorial Policies and the Editorial Policy Checklist.

Statistics

For all statistical analyses, confirm that the following items are present in the figure legend, table legend, main text, or Methods section.

n/a	Confirmed	
☐	☒	The exact sample size (*n*) for each experimental group/condition, given as a discrete number and unit of measurement
☐	☒	A statement on whether measurements were taken from distinct samples or whether the same sample was measured repeatedly
☐	☒	The statistical test(s) used AND whether they are one- or two-sided<br>*Only common tests should be described solely by name; describe more complex techniques in the Methods section.*
☒	☐	A description of all covariates tested
☐	☒	A description of any assumptions or corrections, such as tests of normality and adjustment for multiple comparisons
☐	☒	A full description of the statistical parameters including central tendency (e.g. means) or other basic estimates (e.g. regression coefficient) AND variation (e.g. standard deviation) or associated estimates of uncertainty (e.g. confidence intervals)
☐	☒	For null hypothesis testing, the test statistic (e.g. *F*, *t*, *r*) with confidence intervals, effect sizes, degrees of freedom and *P* value noted<br>*Give P values as exact values whenever suitable.*
☒	☐	For Bayesian analysis, information on the choice of priors and Markov chain Monte Carlo settings
☒	☐	For hierarchical and complex designs, identification of the appropriate level for tests and full reporting of outcomes
☒	☐	Estimates of effect sizes (e.g. Cohen's *d*, Pearson's *r*), indicating how they were calculated

Our web collection on statistics for biologists contains articles on many of the points above.

Software and code

Policy information about availability of computer code

Data collection	Roots were scanned with an Epson Expression 12000XL (Seiko Epson) scanner. ImageJ software was used to measure roots. An Axio Imager 2 (Carl-Zeiss) light microscope and LSM 780 (Carl-Zeiss) confocal laser-scanning microscope were used to take microscopic images. Element 2 (Thermo Fisher Scientific) HR-ICP-MS was used for elemental analysis. A NanoDrop 2000 (NanoDrop) was used for photo-spectral analyses. DNA Microarray Scanner with SureScan High Resolution Technology (Model G2565CA, Agilent Technologies) was used for microarray analysis.
Data analysis	All data analyses were carried out with SigmaPlot 11.0 software and plots prepared with GraphPad Prism software v.9.3.1 (https://www.graphpad.com/).

For manuscripts utilizing custom algorithms or software that are central to the research but not yet described in published literature, software must be made available to editors and reviewers. We strongly encourage code deposition in a community repository (e.g. GitHub). See the Nature Portfolio guidelines for submitting code & software for further information.

Data

Policy information about availability of data

All manuscripts must include a data availability statement. This statement should provide the following information, where applicable:

- Accession codes, unique identifiers, or web links for publicly available datasets
- A description of any restrictions on data availability
- For clinical datasets or third party data, please ensure that the statement adheres to our policy

The raw microarray data generated in this study have been deposited and available in the NCBI Gene Expression Omnibus (GEO) under accession GSE217790

(https://www.ncbi.nlm.nih.gov/geo/query/acc.cgi?acc=GSE217790), while the raw RNA sequencing data have been deposited and available in the European Nucleotide Archive (ENA) under project ID PRJEB65916 (https://www.ebi.ac.uk/ena/browser/view/PRJEB65916). All data generated during this study are included in this published article (and its Supplementary Information File). Source data are provided as a Source Data file.

Research involving human participants, their data, or biological material

Policy information about studies with human participants or human data. See also policy information about sex, gender (identity/presentation), and sexual orientation and race, ethnicity and racism.

Reporting on sex and gender	N/A
Reporting on race, ethnicity, or other socially relevant groupings	N/A
Population characteristics	N/A
Recruitment	N/A
Ethics oversight	N/A

Note that full information on the approval of the study protocol must also be provided in the manuscript.

Field-specific reporting

Please select the one below that is the best fit for your research. If you are not sure, read the appropriate sections before making your selection.

[x] Life sciences [ ] Behavioural & social sciences [ ] Ecological, evolutionary & environmental sciences

For a reference copy of the document with all sections, see nature.com/documents/nr-reporting-summary-flat.pdf

Life sciences study design

All studies must disclose on these points even when the disclosure is negative.

Sample size	We chose the sample size based on available literature in the field and our own experience in previous studies. The sample sizes used for all experiments provided sufficient resolving power to show statistical significance due to homogeneity of plant individuals.
Data exclusions	Samples were excluded from data analysis when strong deviation in plant growth after transferring from pre-culture to treatments could be clearly associated from unintentional damage caused during seedling transfer. Samples determined as outliers according to Grubbs' test (alpha = 0.05) were removed prior to data analysis.
Replication	The number of replication are indicated in the figures or the corresponding figure legends. All experiments were conducted at least twice.
Randomization	All plants were grown in the same growth medium and in the same growth cabinet. The position of agar plates containing plants in the growth cabinets followed a completely randomized design. Random oocytes were selected for injection with cRNA or with mock (water).
Blinding	All the experiments were performed without prior knowledge of the final outcome, and therefore blinding was not applied.

Reporting for specific materials, systems and methods

We require information from authors about some types of materials, experimental systems and methods used in many studies. Here, indicate whether each material, system or method listed is relevant to your study. If you are not sure if a list item applies to your research, read the appropriate section before selecting a response.

Materials & experimental systems

n/a	Involved in the study	
x		Antibodies
x		Eukaryotic cell lines
x		Palaeontology and archaeology
x		Animals and other organisms
x		Clinical data
x		Dual use research of concern
	x	Plants

Methods

n/a	Involved in the study	
x		ChIP-seq
x		Flow cytometry
x		MRI-based neuroimaging

Dual use research of concern

Policy information about dual use research of concern

Hazards

Could the accidental, deliberate or reckless misuse of agents or technologies generated in the work, or the application of information presented in the manuscript, pose a threat to:

No	Yes	
☒	☐	Public health
☒	☐	National security
☒	☐	Crops and/or livestock
☒	☐	Ecosystems
☒	☐	Any other significant area

Experiments of concern

Does the work involve any of these experiments of concern:

No	Yes	
☒	☐	Demonstrate how to render a vaccine ineffective
☒	☐	Confer resistance to therapeutically useful antibiotics or antiviral agents
☒	☐	Enhance the virulence of a pathogen or render a nonpathogen virulent
☒	☐	Increase transmissibility of a pathogen
☒	☐	Alter the host range of a pathogen
☒	☐	Enable evasion of diagnostic/detection modalities
☒	☐	Enable the weaponization of a biological agent or toxin
☒	☐	Any other potentially harmful combination of experiments and agents

Plants

Seed stocks

The following T-DNA tagged and transgenic lines were used in this study: SALK_115548 (hyp1), SALK_009629 (almt1), SALK_016297 and SALK_091930 (lpr1 lpr2), SALK_202530 (crr), SALK_013527 (At4g12980), SALK_006551 (At3g59070), SALK_092983 (At4g17280), SALK_204800 (At5g47530), SALK_110375 (At3g07570), SALK_111270 (At3g61750), SALK_089061 (At5g54830), SALK_205490 (air12), SALK_012348 (At2g04850), proSHR::SHR:GFP (Nakajima et al., 2001), proCYCB1;1::GUS (Ferreira et al., 1994), proWOX5::GFP (Blilou et al., 2005). All A. thaliana T-DNA insertion lines were obtained from the Nottingham Arabidopsis Stock Centre, except the lpr1 [illegible]All constructs for plant expression were generated by GreenGate modular cloning 65. The 2151-bp HYP1 promoter sequence and the 2019-bp of HYP1 open reading frame (without stop codon) were amplified from genomic DNA of Col-0 (CS60000) and the 1212-bp long HYP1 coding sequence (without stop codon) amplified from complementary DNA (cDNA). To drive HYP1 expression in specific cell types, the 2008-bp promoter fragment of BRN1 (At1g33280), 2086-bp of SGN1 (At1g61590), 2309-bp of LPR1 (At1g23010) and 1941-bp of FRO2 (At1g01580) were amplified from genomic DNA of Col-0 (CS60000). Amplification was performed using the Phusion High-Fidelity DNA Polymerase (New England Biolabs) using primers listed in Supplementary Table 3. Promoters, open reading frame and coding sequences were cloned into the GreenGate entry modules pGGA000 and pGGC000, respectively. Correct integration of the fragments into entry modules was verified by restriction digestion reactions and the cloned sequences verified by sequencing. To generate transcriptional reporters and translational GFP fusions, individual entry modules were assembled either into the GreenGate binary vector pGGZ001, including a phosphinotricin or hygromycin resistance cassette for antibiotic selection in plants, or into pFASTR A-G for selection of transgenic plants based on the presence of red fluorescence in seeds. Mutated HYP1M90L, HYP1H174L and HYP1H211L/H278L variants were generated by site-directed mutagenesis using the double-primer method 66 and the primers listed in Supplementary Table 2. The final binary vectors were transformed into the Agrobacterium tumefaciens strain GV3101 (containing the helper plasmid pSOUP if transformed with pGGZ001) and finally transferred to A. thaliana (Col-0; CS60000) plants via the flower dip method 67. Representative transgenic lines for each construct were selected from 6-8 independent transformants and used for analysis. p2 double mutant which was kindly provided by Prof. Dr. Steffen Abel.

Novel plant genotypes

The lines almt1 hyp1, hyp1 crr and lpr1 lpr2 hyp1 were generated by crossing the single almt1 and crr or double lpr1 lpr2 mutants with hyp1. Homozygous double or triple mutant plants were identified by PCR using the primers listed in Supplementary Table 1.

Authentication

N/A
